# VISULYZE-assisted nomogram optimization enhances astigmatism correction accuracy in keratorefractive lenticule extraction: a stratified retrospective analysis

**DOI:** 10.3389/fmed.2026.1837057

**Published:** 2026-07-02

**Authors:** Yanfen Liao, Nian Guan, Bin Zhang, Zhengwei Shen

**Affiliations:** 1 Department of Refractive Surgery, Hubei Bright Eye Hospital, Wuhan, Hubei, China; 2 Department of Refractive Surgery, Wuhan Bright Eye Hospital, Wuhan, Hubei, China

**Keywords:** astigmatism stratification, keratorefractive lenticule extraction, nomogram optimization, refractive surgery, vector analysis, VISULYZE

## Abstract

**Background:**

Keratorefractive lenticule extraction (KLEx) commonly relies on empirical nomogram adjustments to improve refractive accuracy; however, astigmatism correction remains suboptimal. VISULYZE (Carl Zeiss Meditec AG) enables data-driven nomogram optimization, but its differential effectiveness across astigmatism severities has not been fully characterized. This study compared VISULYZE-assisted nomogram optimization with empirical nomogram adjustment for spherical and astigmatic correction, using stratified vector analysis to quantify gains in precision.

**Methods:**

This retrospective case-control study included 206 eyes that underwent KLEx between December 2023 and July 2024 and completed a minimum 3-month follow-up. Eyes were assigned to either VISULYZE-assisted nomogram optimization (VISULYZE group, *n* = 99) or conventional empirical adjustment with a 10% spherical power increment (control group, *n* = 107). Stratified analyses were performed for low-to-moderate astigmatism (< 2.00 D; 57 vs. 62 eyes) and high astigmatism (≥2.00 D; 42 vs. 45 eyes). Primary outcomes included uncorrected distance visual acuity (UDVA), best-corrected distance visual acuity (BCVA), spherical equivalent (SE) accuracy, and residual astigmatism. Astigmatism vector analysis was performed using the Alpins method, including target-induced astigmatism (TIA), surgically induced astigmatism (SIA), difference vector (DV), and correction index (CI). Two-way analysis of variance was used to test interaction effects between nomogram type and astigmatism severity.

**Results:**

At 3 months, 100% of eyes in the VISULYZE group achieved a UDVA of 20/20 or better, compared with 98% of control eyes; furthermore, 87% of the VISULYZE group vs. 73% of the control group achieved 20/16 or better (*P* = 0.045). SE within ±0.50 D was achieved in 100% of VISULYZE eyes and 94.4% of control eyes (*P* = 0.017). Residual astigmatism of 0.50 D or less was observed in 99% vs. 93.5% of eyes, respectively (*P* = 0.042). Stratified analysis showed consistent benefits: both the low-to-moderate and high astigmatism subgroups demonstrated significantly better SE accuracy and lower residual astigmatism with VISULYZE (both *P* < 0.05). Interaction analysis (nomogram type × astigmatism severity) showed no significant effect on any primary outcome (all *P* > 0.05), indicating consistent VISULYZE-associated benefits across strata. Vector analysis showed significantly smaller DV values in VISULYZE eyes across both strata (0.00 vs. 0.25 D, *P* < 0.001; the TIA-SIA correlation was stronger in low-to-moderate astigmatism (R^2^ = 0.9798 vs. 0.9363). Efficacy and safety indices did not differ significantly between groups.

**Conclusion:**

VISULYZE-assisted nomogram optimization improved the accuracy of both spherical and astigmatic correction in KLEx, with consistent benefits across astigmatism severities. This data-driven, iterative refinement paradigm may standardize nomogram development and may be particularly valuable for less experienced surgeons transitioning from empirical adjustment to evidence-based, dynamically optimized surgical planning.

## Introduction

Keratorefractive lenticule extraction (KLEx) has become a mainstream procedure for correcting myopia and astigmatism due to its flapless, minimally invasive design and favorable biomechanical profile (1–5). However, precise postoperative refractive outcomes remain challenging, with 10%-15% of eyes showing spherical equivalent (SE) deviations greater than ±0.50 D after standard small-incision lenticule extraction (SMILE) (6, 7). Astigmatism correction is particularly susceptible to error. Previous studies have reported overall undercorrection rates of approximately 11% (8), with specific rates of 13% per diopter for low astigmatism and 16% per diopter for high astigmatism (9). These discrepancies are primarily attributed to inter-surgeon variability in nomogram adjustments and limited feedback mechanisms. Consequently, adjustments of approximately 10% have been suggested (8, 10), although no standardized nomogram has been established. These errors contribute to patient dissatisfaction (11) and enhancement surgery rates of approximately 1%−2% (12). Accuracy is further reduced in high myopia (13), highlighting the limitations of conventional nomograms and the need for improved predictive models (13, 14).

Nomograms are essential for refining laser input parameters beyond manufacturer defaults (15); however, conventional empirical nomograms rely heavily on surgeon experience and non-standardized adjustment ranges, typically involving 5%−10% incremental modifications (16). Traditional surgical optimization is reliant on surgeon-specific nomograms, which are time-consuming to develop and often fail to account for patient-level variability, thereby compromising accuracy. This subjectivity introduces inconsistency, particularly for astigmatism, in which magnitude-axis coupling requires high precision. Recent advances have incorporated linear regression and machine learning algorithms into nomogram development, with improved predictability (17–21). In 2021, Carl Zeiss Meditec introduced VISULYZE, a software platform that integrates historical surgical data with iterative regression modeling to generate personalized, data-driven nomograms. Emerging evidence indicates that VISULYZE-guided KLEx improves refractive accuracy: Wan et al. (22) reported that 98.1% of eyes were within ±0.50 D of the target SE and that 99.2% had a residual cylinder of 0.50 D or less at 3 months, whereas Shi et al. (23) demonstrated superior astigmatism vector alignment in high-cylinder cohorts (≥1.50 D). Similarly, Lin et al. (24) and Siregar et al. (25) confirmed favorable safety and efficacy profiles with VISULYZE-generated nomograms.

Nevertheless, previous VISULYZE studies have generally analyzed astigmatism outcomes as a single continuum without stratification by severity. This represents an important evidence gap, because low-to-moderate (< 2.00 D) and high (≥2.00 D) astigmatism differ in biomechanical response, vector sensitivity, and clinical tolerance to residual error. In addition, the translational value of VISULYZE as a standardized learning tool for surgeons with different levels of experience remains underexplored.

In the present study, an astigmatism-stratified analytical framework was used to evaluate the differential efficacy of VISULYZE-assisted nomogram optimization. By comparing refractive and vector outcomes between VISULYZE-guided and conventional empirical nomogram groups across both astigmatism severity strata, we aimed to quantify the precision benefits of dynamic, data-driven surgical planning and to establish VISULYZE as a reproducible platform for improving KLEx planning precision and accelerating surgical learning curves.

## Methods

### Study design and ethics

This retrospective case-control study was conducted at Hubei Bright Eye Hospital, Wuhan, China, from December 2023 to July 2024. The study protocol was approved by the Ethics Committee of Hubei Bright Eye Hospital (approval no. 2025-4; approval date: February 6, 2025) and was conducted in accordance with the Declaration of Helsinki. Written informed consent for the use of de-identified clinical data was obtained from all participants prior to inclusion.

### Participants

This study included 206 eyes that underwent KLEx at Hubei Bright Eye Hospital between December 2023 and July 2024 and completed at least 3 months of postoperative follow-up. The inclusion criteria were age of 18 years or older, stable refraction for at least 2 years, estimated residual stromal thickness greater than 250 μm after KLEx, preoperative manifest spherical refraction between −0.50 D and −10.00 D, and manifest cylindrical refraction less than −5.00 D. Patients were required to discontinue contact lens wear for at least 3 months for orthokeratology lenses, 1 month for rigid gas-permeable lenses, and 2 weeks for soft contact lenses prior to surgery. The exclusion criteria were a history of ocular trauma or disease, systemic medical conditions that could affect visual outcomes, and pregnancy or lactation.

Eyes were assigned to a VISULYZE-optimized nomogram group (VISULYZE group, 99 eyes) or a conventional empirical nomogram group (control group, 107 eyes). The VISULYZE nomogram was derived from data from 278 eyes that underwent standard KLEx in 2023. These data were analyzed using VISULYZE software to establish regression models describing the relationships between actual and predicted spherical equivalent (SE) (Y = 0.922 × X; R^2^ = 0.987) and between actual and predicted machine-corrected cylinder (Y = 0.963 × X; R^2^ = 0.968). Based on these models, a customized nomogram was developed to guide laser parameter selection. Conversely, the control group was treated using a conventional experience-based nomogram, wherein a 10% increase in spherical correction was applied without adjusting cylinder parameters.

For the stratified analysis, eyes were categorized according to the magnitude of preoperative refractive astigmatism into low-to-moderate astigmatism (< 2.00 D; 57 VISULYZE vs. 62 control eyes) and high astigmatism (≥2.00 D; 42 VISULYZE vs. 45 control eyes).

### Perioperative management

Preoperatively, all patients were administered topical levofloxacin eye drops four times daily for 3 days. Postoperatively, patients were treated with 0.5% levofloxacin ophthalmic solution (Santen, Japan) four times daily for 1 week. Topical fluorometholone (Santen, Japan) was prescribed with a gradual tapering schedule over 1 month. Artificial tears (0.1% sodium hyaluronate ophthalmic solution; HYLO COMOD, Ursapharm, Saarbrücken, Germany) were administered four times daily for 3 months postoperatively.

### Surgical procedures

All KLEx procedures were performed using the VisuMax 500 femtosecond laser system (Carl Zeiss Meditec AG, Jena, Germany). Surgical parameters included a cap diameter of 7.5–7.8 mm, a 2-mm incision positioned at 120°, an optical zone of 6.5–6.8 mm with a 0.1-mm transition zone for astigmatism correction, and a cap thickness of 110–120 μm. Laser parameters for lenticule creation were set at a pulse energy of 130 nJ, with spot and track spacings of 4.5 μm. The laser delivery sequence began with the posterior lenticule cut, followed by the anterior lenticule cut and subsequently the side cut. With the curved contact glass in place, the patient was instructed to fixate on a blinking target. After proper centration was confirmed, suction was activated and laser emission was initiated. The corneal incision was then opened. A thin blunt spatula was used to separate the superficial and deep planes of the lenticule, disrupt any residual tissue bridges, and free the lenticule from the surrounding stroma. The lenticule was subsequently grasped with forceps and gently withdrawn through the 2-mm incision. All procedures were performed by a single surgeon (Z.W.S.) possessing over 30 years of experience in corneal refractive surgery.

### Preoperative and postoperative examinations

Comprehensive ophthalmic examinations were performed preoperatively and at 1 and 3 months postoperatively. Assessments included slit-lamp biomicroscopy, fundus examination, measurements of uncorrected distance visual acuity (UDVA) and best-corrected distance visual acuity (BCVA), manifest refraction, and anterior segment tomography using the Pentacam HR system (Oculus Optikgeräte GmbH, Wetzlar, Germany). Visual acuity measurements were converted to the logMAR scale for statistical analysis.

Surgical outcomes at 3 months were assessed using the standard nine-graph reporting system (26), with report charts generated by VISULYZE software. Primary outcome measures included UDVA, BCVA, residual spherical error, residual cylindrical error, and SE. The efficacy and safety indices were calculated as the ratio of postoperative UDVA to preoperative BCVA and the ratio of postoperative BCVA to preoperative BCVA, respectively.

Astigmatism correction was evaluated using the Alpins vector analysis method (27), utilizing the ASSORT online calculator provided by the International Refractive Surgery Society for calculations. The evaluated parameters included angle of error, correction index (CI), surgically induced astigmatism (SIA), target-induced astigmatism (TIA), and difference vector (DV), which represents the magnitude of residual astigmatism after surgery.

### Statistical analysis

Statistical analyses were performed using SPSS software (version 25.0; IBM SPSS Inc., Chicago, IL, USA). Data normality was assessed using the Kolmogorov-Smirnov test. Normally distributed continuous variables were expressed as the mean ± standard deviation, whereas non-normally distributed variables were expressed as the median (Q1, Q3). Categorical variables were presented as frequencies or percentages.

Baseline characteristics were compared between groups using chi-square tests for categorical variables and independent-samples *t* tests or Mann-Whitney U tests for continuous variables, depending on data distribution and homogeneity of variance.

For between-group comparisons of outcomes, independent-samples *t* tests were used for normally distributed data with homogeneity of variance, whereas Mann-Whitney U tests were used for non-normally distributed or heteroscedastic data. To evaluate whether the effect of nomogram type (VISULYZE vs. conventional) varied according to astigmatism severity, we tested the nomogram × astigmatism-stratum interaction for all primary and secondary outcomes. Specifically, two-way ANOVA or linear mixed models were used for continuous variables, while the Cochran-Mantel-Haenszel test was employed for categorical variables. Non-significant interaction terms (*P* > 0.05) were interpreted as suggesting consistent nomogram effects across strata. When significant interactions were identified, simple-effect analyses were performed to characterize the differential efficacy of VISULYZE nomograms within each stratum.

A two-sided *P* value < 0.05 was considered statistically significant for all analyses.

## Results

### Baseline characteristics

The study cohort comprised 206 eyes, with 99 assigned to the VISULYZE group and 107 to the control group. Baseline characteristics stratified by astigmatism severity are presented in Table 1. In the low-to-moderate astigmatism stratum (< 2.00 D), the VISULYZE group exhibited a significantly greater preoperative cylinder magnitude than the control group (−1.00 ± 0.47 D vs. −0.74 ± 0.43 D, *P* = 0.002). A modest but statistically significant age difference was also observed between the groups (24.56 ± 5.32 years vs. 27.08 ± 7.60 years, P = 0.040). No significant between-group differences were found for sex distribution, spherical power, or spherical equivalent (SE) (all *P* > 0.05). In the high astigmatism stratum (≥2.00 D), all baseline characteristics were evenly distributed between the two groups (all *P* > 0.05).

**Table 1:** Baseline demographic and preoperative characteristics by astigmatism severity.

Astigmatism stratum	Variable	Eyes, *n*	Age (years)	Sex (M/F)	Sphere (D), mean ±SD (range)	Cylinder (D), mean ±SD (range)	SE (D), mean ±SD (range)
Low-to-moderate astigmatism group	VISULYZE group	57	24.56 ± 5.32 (18, 37)	29/28	−4.39 ± 1.62 (−7.75, −1)	−1 ± 0.47 (−1.75, 0)	−4.89 ± 1.62 (−8.13, −1.25)
	Control group	62	27.08 ± 7.6 (18, 41)	30/32	−4.14 ± 1.69 (−8.5, −1.25)	−0.74 ± 0.43 (−1.75, 0)	−4.51 ± 1.71 (−8.63, −1.63)
	*P* value		0.04^a^	0.785^b^	0.406^a^	0.002^a^*	0.215^a^
High Astigmatism group	VISULYZE group	42	22.88 ± 4.98 (18, 35)	26/16	−4.24 ± 2.13 (−7.25, −0.75)	−2.55 ± 0.64 (−4.25, −2)	−5.52 ± 1.99 (−8.5, −1.75)
	Control group	45	24.24 ± 7.54 (18, 48)	29/16	−4.42 ± 1.69 (−7.25, −0.25)	−2.61 ± 0.52 (−3.75, −2)	−5.72 ± 1.62 (−8.25, −1.75)
	*P* value		0.326^a^	0.531^b^	0.674^a^	0.643^a^	0.601^a^

SD, standard deviation; D, diopter; SE, spherical equivalent.

Data are presented as mean ± standard deviation or frequency. For between-group comparisons, continuous variables with normal distribution and homogeneity of variance were analyzed using independent-samples *t* tests; categorical variables were analyzed using chi-square tests. ^*^P < 0.05. ^a^, Statistical test: independent-samples *t* test; ^b^, Statistical test: chi-square test.

### Overall refractive outcomes

**Figure 1:**
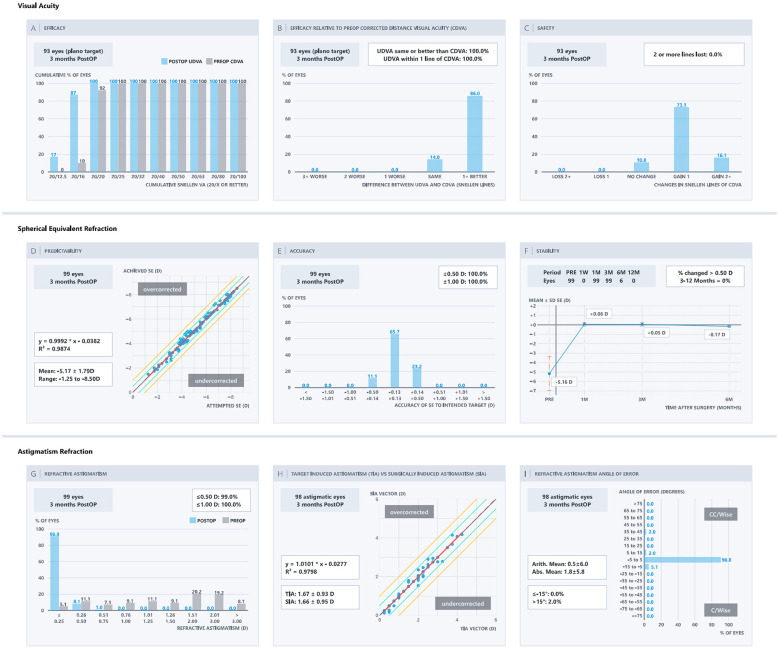
Refractive surgery outcomes at 3 months in the VISULYZE group. Standardized outcome measures at the 3-month postoperative follow-up: **(A)** Efficacy, depicted as uncorrected distance visual acuity (UDVA). **(B)** Comparison between UDVA and best-corrected visual acuity (BCVA). **(C)** Safety, assessed by changes in BCVA. **(D)** Predictability, demonstrated by the relationship between attempted and achieved spherical equivalent (SE) correction. **(E)** Accuracy of postoperative refractive outcomes based on SE. **(F)** Stability, illustrated by temporal changes in SE refraction. **(G)** Residual postoperative astigmatism. **(H)** Predictability of astigmatism correction, comparing attempted vs. achieved astigmatism correction. **(I)** Angle of error for refractive astigmatism.

**Figure 2:**
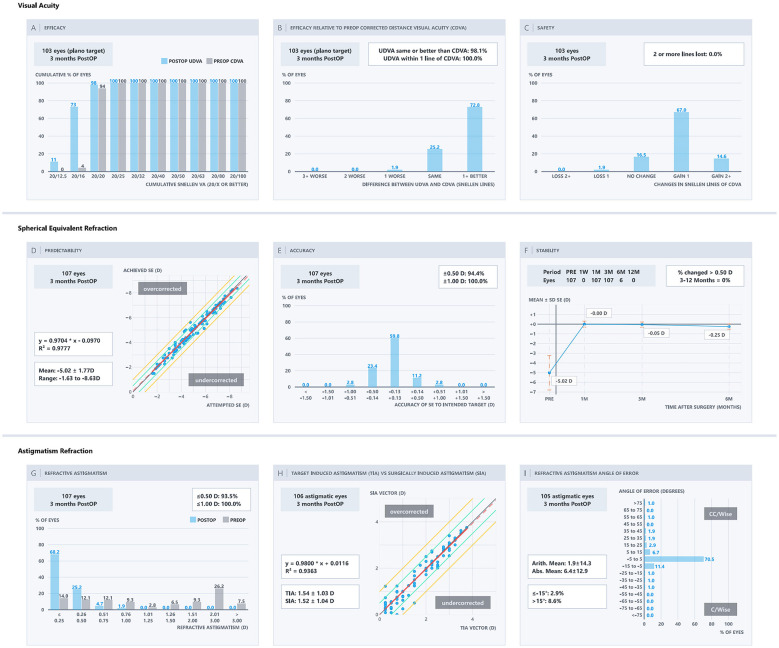
Refractive surgery outcomes at 3 months in the control group. Standardized outcome graphs at the 3-month postoperative follow-up: **(A)** Efficacy, depicted by uncorrected distance visual acuity (UDVA). **(B)** Comparison between UDVA and best-corrected visual acuity (BCVA). **(C)** Safety, assessed by changes in BCVA. **(D)** Predictability based on the relationship between attempted and achieved spherical equivalent (SE) correction. **(E)** Accuracy of postoperative refractive outcomes according to SE. **(F)** Stability, illustrated by temporal changes in SE refraction. **(G)** Residual postoperative astigmatism. **(H)** Predictability of astigmatism correction, comparing attempted vs. achieved correction. **(I)** Angle of error for refractive astigmatism.

Standardized nine-graph refractive outcome reports for the VISULYZE and control groups are shown in Figures 1 and 2, respectively. At 3 months postoperatively, all eyes in the VISULYZE group achieved uncorrected distance visual acuity (UDVA) of 20/20 or better, and 87% achieved 20/16 or better (Figure 1A). In the control group, 98% achieved 20/20 or better and 73% achieved 20/16 or better (Figure 2A). Postoperative UDVA was equal to or better than preoperative corrected distance visual acuity (CDVA) in 100% of the VISULYZE group (Figure 1B) and 98.1% of the control group (Figure 2B). No eye in the VISULYZE group lost any lines of CDVA (Figure 1C), compared with 1.9% in the control group (Figure 2C). Mean postoperative UDVA was significantly better in the VISULYZE group than in the control group (−0.11 ± 0.01 logMAR vs. −0.09 ± 0.01 logMAR, *P* = 0.023).

Scatterplots of attempted vs. achieved SE showed high predictability in both groups (VISULYZE: R^2^ = 0.9874; control: R^2^ = 0.9777) (Figures1D, 2D). The proportion of eyes within ±0.50 D of the target SE was 100% in the VISULYZE group and 94.4% in the control group (Figures1E, 2E; *P* = 0.017). Residual astigmatism of 0.50 D or less was achieved in 99% of VISULYZE eyes and 93.5% of control eyes.

### Stratified refractive outcomes by astigmatism severity

Refractive outcomes stratified by astigmatism severity are summarized in Table 2. Interaction analysis showed no significant interaction between the nomogram type and the astigmatism stratum for any outcome measure (all *P* > 0.05), indicating consistent benefits of VISULYZE across the astigmatism spectrum.

**Table 2:** Refractive outcomes at the 3-month postoperative follow-up.

Astigmatism stratum	Parameter	VISULYZE group (mean ±SD, range)	Control group (mean ±SD, range)	*P* value
Low-to-moderate astigmatism group	UDVA, logMAR	−0.116 ± 0.056 (−0.2, 0)	−0.093 ± 0.068 (−0.2, 0.1)	0.045^*^
	BCVA, logMAR	−0.124 ± 0.054 (−0.2, 0)	−0.111 ± 0.067 (−0.2, 0.1)	0.315
	Sphere, D	0.11 ± 0.164 (0, 0.5)	0.125 ± 0.303 (−0.5, 1)	0.614
	Cylinder, D	−0.057 ± 0.125 (−0.5, 0)	−0.25 ± 0.268 (−1, 0)	< 0.001^*^
	SE, D	0.081 ± 0.172 (−0.13, 0.5)	0 ± 0.309 (−0.63, 0.88)	0.025^*^
	Efficacy index	1.278 ± 0.173 (1, 1.6)	1.236 ± 0.202 (0.8, 1.6)	0.128
	Safety index	1.3 ± 0.175 (1, 1.6)	1.287 ± 0.201 (0.8, 1.6)	0.642
High Astigmatism group	UDVA, logMAR	−0.086 ± 0.045 (−0.2, 0)	−0.076 ± 0.051 (−0.2, 0)	0.337
	BCVA, logMAR	−0.088 ± 0.043 (−0.2, 0)	−0.076 ± 0.046 (−0.2, 0)	0.194
	Sphere, D	0.077 ± 0.187 (−0.25, 0.5)	0.033 ± 0.189 (−0.5, 0.5)	0.278
	Cylinder, D	−0.161 ± 0.205 (−0.75, 0)	−0.272 ± 0.219 (−0.75, 0)	0.016^*^
	SE, D	−0.004 ± 0.19 (−0.25, 0.5)	−0.104 ± 0.209 (−0.63, 0.38)	0.022^*^
	Efficacy index	1.267 ± 0.149 (1, 1.6)	1.238 ± 0.169 (1, 1.6)	0.404
	Safety index	1.271 ± 0.129 (1, 1.6)	1.236 ± 0.156 (1, 1.6)	0.253

UDVA, uncorrected distance visual acuity; BCVA, best corrected visual acuity; SE, spherical equivalent; D, diopters.

Data are presented as mean ± standard deviation. For between-group comparisons, continuous variables with normal distribution and homogeneity of variance were analyzed using independent-samples *t* tests. ^*^P < 0.05.

#### Low-to-moderate astigmatism stratum

The VISULYZE group demonstrated significantly better UDVA (−0.116 ± 0.056 logMAR vs. −0.093 ± 0.068 logMAR, *P* = 0.045) and lower residual cylinder (−0.057 ± 0.125 D vs. −0.250 ± 0.268 D, *P* < 0.001) than the control group. SE accuracy was also superior in the VISULYZE group (0.081 ± 0.172 D vs. 0.000 ± 0.309 D, *P* = 0.025). The efficacy and safety indices did not differ significantly between the groups (*P* = 0.128 and *P* = 0.642, respectively).

#### High astigmatism stratum

The VISULYZE group showed significantly lower residual cylinder (−0.161 ± 0.205 D vs. −0.272 ± 0.219 D, *P* = 0.016) and more accurate SE (−0.004 ± 0.19 D vs. −0.104 ± 0.209 D, *P* = 0.022) than the control group. UDVA and the efficacy and safety indices did not differ significantly between groups (all *P* > 0.05).

### Astigmatism vector analysis

**Figure 3:**
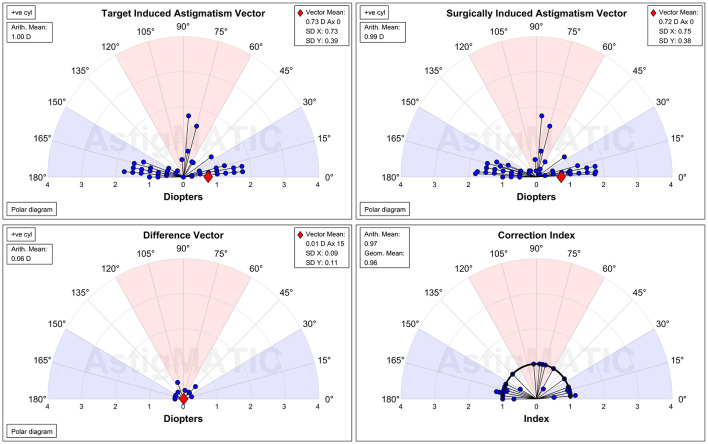
Vector analysis of refractive cylinder in the low-to-moderate astigmatism VISULYZE group. Double-angle vector plots illustrating postoperative refractive astigmatism at 3 months, including target-induced astigmatism, surgically induced astigmatism, difference vector, and correction index.

**Figure 4:**
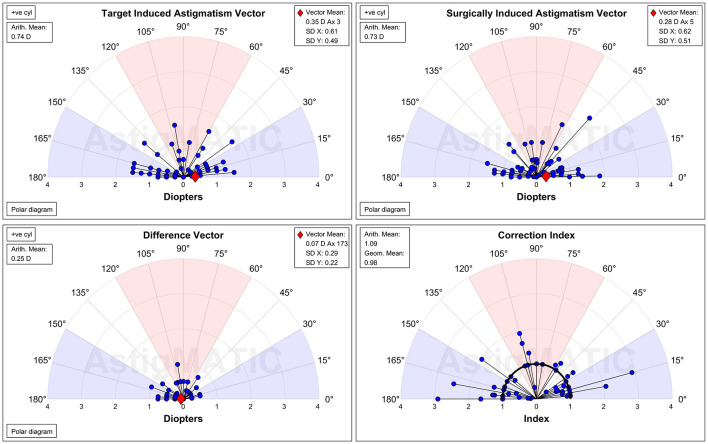
Vector analysis of refractive cylinder in the low-to-moderate astigmatism control group. Double-angle vector plots illustrating postoperative refractive astigmatism at 3 months, including target-induced astigmatism, surgically induced astigmatism, difference vector, and correction index.

**Figure 5:**
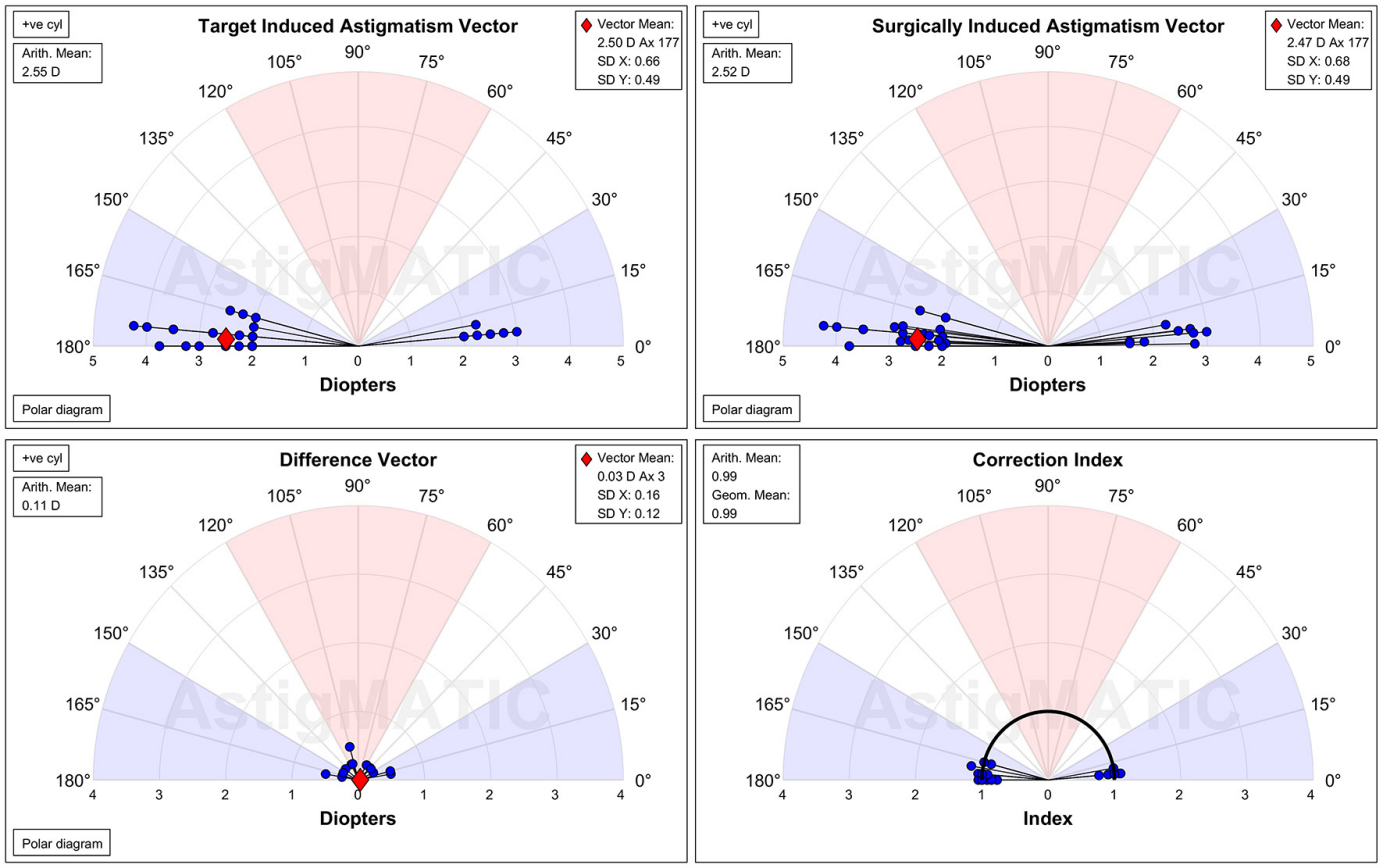
Vector analysis of refractive cylinder in the high astigmatism VISULYZE group. Double-angle vector plots illustrating postoperative refractive astigmatism at 3 months, including target-induced astigmatism, surgically induced astigmatism, difference vector, and correction index.

**Figure 6:**
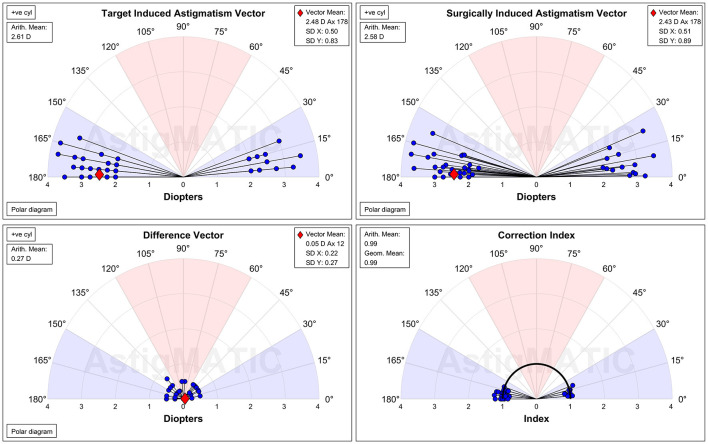
Vector analysis of refractive cylinder in the high astigmatism control group. Double-angle vector plots illustrating postoperative refractive astigmatism at 3 months, including target-induced astigmatism, surgically induced astigmatism, difference vector, and correction index.

Astigmatism outcomes evaluated using the Alpins vector analysis method are shown in Figures 3, 4, 5, 6 and Table 3; Table 3 specifically presents vector analysis results stratified by astigmatism severity.

**Table 3:** Astigmatism vector analysis outcomes at 3-month postoperative follow-up.

Astigmatism stratum	Parameter	VISULYZE group [Median (Q1, Q3) (range)]	Control group [Median (Q1, Q3) (range)]	*P* value
Low-to-moderate astigmatism group	TIA, D	1(0.5,1.38) (0, 1.75)	0.75(0.44, 1) (0, 1.75)	0.003^*^
	SIA, D	1(0.5, 1.38) (0.13, 1.8)	0.64(0.49, 1) (0, 2.13)	0.002^*^
	DV, D	0(0, 0) (0, 0.5)	0.25(0, 0.5) (0, 1)	< 0.001^*^
	CI	1(1, 1) (0.35, 1.2)	1(0.86, 1.06) (0, 4.89)	0.965
High astigmatism group	TIA, D	2.25(2, 2.81) (2, 4.25)	2.5(2.25, 3) (2, 3.75)	0.276
	SIA, D	2.3(2, 2.77) (1.54, 4.25)	2.5(2.14, 2.96) (1.72, 3.75)	0.327
	DV, D	0(0, 0.25) (0, 0.5)	0.25(0, 0.5) (0, 0.75)	< 0.001^*^
	CI	1(1, 1) (0.77, 1.18)	1(0.93, 1.02) (0.8, 1.25)	0.699

CI, correction index; DV, difference vector; SIA, surgically induced astigmatism; TIA, target-induced astigmatism; D, diopter

Non-normally distributed variables are presented as median (Q1, Q3) and were compared using the Mann-Whitney U test. ^*^Statistically significant.

#### Low-to-moderate astigmatism stratum

The VISULYZE group exhibited significantly higher target-induced astigmatism (TIA) (1.00 D vs. 0.75 D, *P* = 0.003) and surgically induced astigmatism (SIA) (1.00 D vs. 0.64 D, *P* = 0.002), suggesting more precise magnitude-axis coupling. The difference vector (DV) was significantly smaller in the VISULYZE group (0.00 D vs. 0.25 D, *P* < 0.001), indicating superior vector accuracy. No significant differences were observed in the correction index (CI) between the groups (1.00 vs. 1.00, *P* = 0.965).

#### High astigmatism stratum

The VISULYZE group demonstrated a significantly smaller DV than the control group (0.00 D vs. 0.25 D, *P* < 0.001). No significant differences were observed in TIA, SIA, or CI between the groups (*P* = 0.276, *P* = 0.327, and *P* = 0.699, respectively).

## Discussion

This study demonstrates that VISULYZE-assisted nomogram optimization results in superior surgical outcomes compared with conventional KLEx, particularly for astigmatism correction. The primary finding, specifically a significant reduction in residual cylinder and spherical equivalent deviation across both low-to-moderate and high astigmatism strata, supports VISULYZE as a reproducible, data-driven surgical planning platform that offers an advantage over traditional, experience-dependent nomogram adjustments. Notably, this study introduces an astigmatism-stratified analytical framework for evaluating VISULYZE-assisted optimization, representing a methodological advance that differentiates this study from previous VISULYZE publications.

### Efficacy and refractive precision

KLEx is well established for its high efficacy, safety, and predictability (28–31). Our findings are consistent with previous reports on VISULYZE-assisted parameter selection, which demonstrated excellent correction accuracy and safety at 3 months postoperatively (22–25). Both groups exhibited excellent safety profiles, with no significant differences in complications, confirming that VISULYZE-based refractive parameter design maintains surgical safety while incorporating an innovative planning strategy.

Notably, VISULYZE-assisted KLEx yielded superior mean postoperative UDVA; the proportion of eyes achieving UDVA of 20/16 or better at 3 months was 14 percentage points higher than that after conventional KLEx (87% vs. 73%). Correction accuracy was also higher, with a 5.6-percentage-point increase in the proportion of eyes achieving postoperative residual SE within ±0.50 D (100% vs. 94.4%). Together, these advantages reflect the more comprehensive refractive assessment and reduced planning error afforded by data-driven planning, indicating that VISULYZE improves both the efficacy and precision of KLEx.

These superior outcomes may be attributable to the platform's fundamental shift from experience-driven to data-driven surgical planning. VISULYZE integrates historical surgical data with machine learning to generate individualized nomograms. Unlike systems that rely on fixed algorithms (32) or lack dynamic learning capabilities (33), VISULYZE uses dynamic feedback and iterative refinement to enable outcome-driven adjustment. This approach mitigates human error and enables more comprehensive refractive assessment through real-time optimization, thereby improving surgical planning precision while accommodating inter-surgeon variability. In addition, the platform's outcome visualization feature allows surgeons to simulate and refine plans preoperatively, enhancing decision-making.

### Astigmatism-stratified precision: addressing a critical gap in VISULYZE evidence

Precise astigmatism correction remains challenging in KLEx, with previous studies reporting a tendency toward undercorrection, particularly in eyes with higher preoperative astigmatism (8, 9, 34, 35). Notably, the present study introduces a prespecified astigmatism-stratified analytical framework to evaluate VISULYZE-assisted nomogram optimization, representing a methodological advance that distinguishes this work from previous VISULYZE publications. Wan et al. (22) first reported outcomes of VISULYZE-generated nomograms in an unstratified cohort of 260 eyes, demonstrating 98.1% predictability for SE within ±0.50 D and indicating that 99.2% of eyes achieved a residual cylinder of 0.50 D or less at 3 months. Although these findings established proof of concept for data-driven KLEx planning, the absence of astigmatism severity stratification restricted their clinical specificity.

Specifically, in the low-to-moderate astigmatism stratum (< 2.00 D), a significantly better UDVA (−0.116 ± 0.056 logMAR vs. −0.093 ± 0.068 logMAR, *P* = 0.045) was observed, accompanied by a markedly lower residual cylinder (−0.057 ± 0.125 D vs.−0.25 ± 0.268 D, *P* < 0.001). These stratified results build upon the unstratified outcomes reported by Wan et al. (36) (98.9% vs. 85.6% within ±0.50 D SE, *P* < 0.001) by demonstrating that the precision advantage is particularly evident in eyes with lower baseline cylinders. The mechanism may involve the VISULYZE dynamic regression algorithm detecting subtle undercorrection trends that are historically masked in pooled data and compensating through refined target adjustments. This interpretation is consistent with Li et al. (37), who reported that the VISULYZE quadratic regression model (y = 0.0077 x 2 + 0.9775 x, R^2^ = 0.996) outperformed linear empirical nomograms in SE accuracy (97% vs. 81% within ±0.50 D).

In contrast, the high astigmatism stratum (≥2.00 D) showed comparable UDVA between groups (*P* = 0.337), despite significant improvements in SE accuracy (*P* = 0.022) and residual cylinder (*P* = 0.016). This pattern differs from that reported by Shi et al. (23), who found 100% residual cylinder of 0.50 D or less in high astigmatism (≥1.50 D) with VISULYZE, compared with 88.9% in controls. This discrepancy may reflect differences in cohort characteristics: Shi's VISULYZE group had a lower baseline cylinder (−1.75 D median vs. −2.55 ± 0.64 D in our cohort), suggesting a dose-dependent optimization benefit with greater absolute improvements in more severe astigmatism. Alternatively, the conventional 10% spherical increment used in our control group, without cylinder adjustment, may have introduced compensatory errors that partly masked the visual acuity advantage of VISULYZE in this stratum. This finding underscores the value of stratified reporting, because aggregated analyses may obscure clinically meaningful heterogeneity that informs surgical planning.

### Vector analysis: precision mechanisms and comparative insights

Alpins vector analysis revealed a consistent pattern across strata, highlighting the mechanistic distinction between VISULYZE and conventional nomograms. In both strata, difference vectors were significantly smaller in the VISULYZE groups (low-to-moderate: 0 D vs. 0.25 D, *P* < 0.001; high: 0 D vs. 0.25 D, *P* < 0.001), indicating improved magnitude-axis coupling precision. However, correction indices remained comparable (all *P* > 0.05), suggesting that VISULYZE enhances targeting accuracy without altering the fundamental correction ratio of the laser platform.

This dissociation has important implications when considered alongside the findings of Siregar et al. (25), who combined VISULYZE nomograms with OcuLign cyclotorsion alignment and reported a correction index of 0.992. Our CI values (1.00 in both VISULYZE groups) are consistent with this benchmark, and notably, these results were achieved without rotational compensation. This comparison suggests that the primary contribution of VISULYZE lies in refining the planning phase (target vector precision), whereas OcuLign addresses the execution phase (axis alignment). The additive potential of combining these technologies, which was not tested in our cohort, represents a promising direction for future investigation.

The elevated TIA and SIA in the low-to-moderate VISULYZE group (1.00 D vs. 0.75 D, *P* = 0.003; 1.00 D vs. 0.64 D, *P* = 0.002) require careful interpretation. Unlike Wan et al. (22), who reported TIA/SIA correlations without stratification, our stratified analysis shows that the VISULYZE algorithm actively increases cylindrical targets in response to detected undercorrection patterns. This dynamic adjustment contrasts with static empirical nomograms that apply fixed incremental modifications regardless of outcome feedback. Lin et al. (24) similarly observed that VISULYZE-generated nomograms improved predictability (R^2^ = 0.9977) compared with conventional planning, although their study focused on myopic astigmatism without severity stratification. Our findings extend the observations of Lin et al. by demonstrating that the adaptive behavior of the algorithm varies across astigmatism strata, with greater target augmentation in lower cylinder magnitudes, where subtle residual errors may disproportionately affect visual quality.

### VISULYZE as a dynamic learning platform: clinical translation and educational value

A key contribution of this study is the positioning of VISULYZE not merely as a nomogram calculator but as a dynamic learning system that addresses important unmet needs in refractive surgery standardization. Conventional nomogram development relies on tacit knowledge accumulated over years of practice, creating substantial barriers for novice surgeons and perpetuating inter-practitioner variability (16, 38). Liang et al. (16) documented that surgeon-specific nomogram adjustments range from 5% to 10%, reflecting this non-standardized variability. VISULYZE transforms this opaque process into a transparent, data-driven protocol with iterative feedback capability.

For training programs, VISULYZE offers three distinct advantages over traditional apprenticeship models. First, it provides evidence-based starting parameters derived from institutional historical data, reducing the initial case volume required to achieve competency. Wan et al. (36) observed that VISULYZE's automated regression modeling eliminates the manual data aggregation and statistical analysis previously required for nomogram construction. Second, the software outcome visualization tools enable real-time performance monitoring, allowing supervisors to detect subtle refractive drift in trainees before it becomes clinically significant. Third, the iterative refinement loop in VISULYZE, which integrates new case data to continuously update regression coefficients, creates a self-improving system that accelerates the transition from competency to mastery (39).

For experienced surgeons, our data demonstrate that VISULYZE provides value beyond the learning curve. Despite more than 30 years of KLEx experience, the single surgeon in this study achieved measurable precision gains through algorithmic optimization. This finding challenges the notion that empirical nomogram refinement reaches a performance ceiling (15). Instead, VISULYZE's capacity to detect subtle systematic errors—often invisible to conventional outcome tracking due to cognitive bias and memory limitations—suggests that data-driven feedback complements, rather than replaces, clinical expertise. This interpretation is consistent with Luft et al. (39), who concluded that artificial intelligence-enhanced nomograms achieved predictability comparable to or superior to that of surgeon-optimized protocols.

## Limitations and future perspectives

Several limitations should be acknowledged. First, the retrospective design introduces temporal confounding, although sequential implementation mirrors real-world VISULYZE adoption patterns and enhances external validity. Second, the single-center, single-surgeon design limits generalizability while strengthening internal validity. Third, baseline cylinder imbalance (*P* = 0.003) and a modest age difference (*P* = 0.040) were observed in the low-to-moderate astigmatism stratum; while sensitivity analyses confirmed the robustness of the findings, this initial imbalance may have amplified the observed UDVA differences. Fourth, the 3-month follow-up precludes definitive conclusions regarding stability, particularly as noted by Siregar et al. (25), who observed that refractive drift may emerge beyond this time frame. Fifth, the absence of subjective visual quality metrics, deliberately excluded due to retrospective design constraints, represents a scope limitation that should be addressed in prospective studies using validated instruments, such as the Quality of Vision questionnaire and the NEI-RQL.

Future research should focus on three key areas: prospective randomized trials with strict unilateral-eye inclusion to eliminate inter-eye correlation; integration of VISULYZE with cyclotorsion compensation and iris-registration systems to evaluate additive precision benefits; and development of astigmatism-specific nomogram algorithms that account for axis-dependent biomechanical responses, building on the stratified insights established herein.

## Conclusions

This astigmatism-stratified analysis demonstrates that VISULYZE-assisted nomogram optimization consistently improves refractive accuracy across all magnitudes of astigmatism in KLEx. Alpins vector analysis demonstrated that the platform enhances magnitude-axis coupling precision without altering the fundamental correction ratio. By converting empirically derived adjustments into a scientific, data-driven, and dynamically iterative system, VISULYZE facilitates more reproducible surgical planning and may shorten the learning curve for novice surgeons, ultimately enhancing overall refractive visual quality. The stratified analytical framework established herein provides a robust template for future precision-medicine approaches in corneal refractive surgery.

## Data Availability

The original contributions presented in the study are included in the article/supplementary material, further inquiries can be directed to the corresponding author.
